# Influence of the trajectory of the urine output for 24 h on the occurrence of AKI in patients with sepsis in intensive care unit

**DOI:** 10.1186/s12967-021-03190-w

**Published:** 2021-12-20

**Authors:** Luming Zhang, Fengshuo Xu, Didi Han, Tao Huang, Shaojin Li, Haiyan Yin, Jun Lyu

**Affiliations:** 1 Intensive Care Unit, The First Affiliated Hospital of Jinan University, Guangzhou, 510630 Guangdong Province People’s Republic of China; 2 Department of Clinical Research, The First Affiliated Hospital of Jinan University, Guangzhou, 510630 Guangdong Province People’s Republic of China; 3 School of Public Health, Xi’an Jiaotong University Health Science Center, Xi’an, Shaanxi China; 4 Department of Orthopaedics, The First Affiliated Hospital of Jinan University, Guangzhou, Guangdong Province China

**Keywords:** Sepsis, Urine output, Acute kidney injury, Latent growth mixture modeling, Doubly robust estimation

## Abstract

**Background:**

Sepsis-associated acute kidney injury (S-AKI) is a common and life-threatening complication in hospitalized and critically ill patients. This condition is an independent cause of death. This study was performed to investigate the correlation between the trajectory of urine output within 24 h and S-AKI.

**Methods:**

Patients with sepsis were studied retrospectively based on the Medical Information Mart for Intensive Care IV. Latent growth mixture modeling was used to classify the trajectory of urine output changes within 24 h of sepsis diagnosis. The outcome of this study is AKI that occurs 24 h after sepsis. Cox proportional hazard model, Fine–Gray subdistribution proportional hazard model, and doubly robust estimation method were used to explore the risk of AKI in patients with different trajectory classes.

**Results:**

A total of 9869 sepsis patients were included in this study, and their 24-h urine output trajectories were divided into five classes. The Cox proportional hazard model showed that compared with class 1, the HR (95% CI) values for classes 3, 4, and 5 were 1.460 (1.137–1.875), 1.532 (1.197–1.961), and 2.232 (1.795–2.774), respectively. Competing risk model and doubly robust estimation methods reached similar results.

**Conclusions:**

The trajectory of urine output within 24 h of sepsis patients has a certain impact on the occurrence of AKI. Therefore, in the early treatment of sepsis, close attention should be paid to changes in the patient's urine output to prevent the occurrence of S-AKI.

**Supplementary Information:**

The online version contains supplementary material available at 10.1186/s12967-021-03190-w.

## Introduction

Sepsis 3.0 defines sepsis as a life-threatening organ dysfunction caused by the host’s dysfunctional response to infection. Sepsis is one of the most common critical diseases in the emergency department and intensive care unit (ICU) [1]. According to reports, the number of patients diagnosed with sepsis worldwide in 2017 was as high as 48.9 million, of which 11 million patients died, accounting for 19.7% of the total deaths in the world, causing a great health burden [2]. Sepsis-associated acute kidney injury (S-AKI) is a common and life-threatening complication in hospitalized and critically ill patients. This condition is an independent cause of death, with septic kidney injury occurring in 50–70% of cases in the ICU [3, 4]. S-AKI is characterized by sepsis accompanied by the rapid deterioration of renal function. This complication is difficult to treat and has high mortality rate, which greatly consumes public health resources [5].

Up to now, a large number of studies on sepsis have deepened the understanding of the risk factors, early warning markers and pathobiological mechanisms of S-AKI. Zhang [6] mentioned in a narrative review that neutrophil gelatinase-associated lipocalin (NGAL), cystatin C, b2-microglobulin and microalbuminuria are potential biomarkers that can predict the occurrence and development of S-AKI. In addition, there are many published papers on serum biomarkers indicating that heparin-binding protein, interleukin-6 (IL-6), interleukin-8 (IL-8) etc. have a good predictive effect on AKI [7–10]. However, these biomarkers still need a lot of prospective studies and trials to prove, and there is still a gap in clinical application and promotion [11].Most of the existing AKI diagnoses are based on the Kidney Disease: Improving Global Outcomes (KDIGO) Clinical Practice Guidelines [12], in which diagnosis and staging are based on changes in the urine output and creatinine. The change in the urine output plays a very important role in the development of AKI. Oliguria for more than 12 h and for 3 or more instances have been associated with increased mortality [13]. Another study has used deep learning methods to continuously predict the occurrence of severe AKI based on changes in the urine output in critically ill patients [14]. However, the effect of the trajectory of the urine output on the occurrence of AKI in patients with sepsis has not been investigated.

In latent growth mixture modeling (LGMM), the population is assumed to be heterogeneous and composed of several latent classes of subjects characterized by a number of mean profiles of trajectories [15]. In this study, we used the LGMM model to classify the 24-h urine output change trajectory of sepsis patients from the large public database Medical Information Mart for Intensive Care IV (MIMIC-IV). We also investigated the influence of sepsis with different trajectories of changes in the urine output on the occurrence of AKI to provide a basis for clinical treatment.

## Methods

### Data source

The MIMIC-IV database is a large, openly accessible, and relational database [16, 17]. It contains a comprehensive information on more than 250,000 electronic admission records of the Beth Israel Deaconess Medical Center in Boston, Massachusetts from 2008 to 2019. These records include the diagnosis, vital signs, laboratory tests, medication, and surgical information [18, 19]. The data used in this study were from the latest version of MIMI-IV 1.0, which was released in March 2021.

After completing the online course of the National Institutes of Health and passing the examination for the protection of human study participants, we were qualified to use the MIMI-IV database (Record ID: 38601114;38455175).

### Study population

We determined the study population based on the Sepsis 3.0 criteria, that is, on the basis of suspected or confirmed infection plus the Sequential Organ Failure Assessment (SOFA) score with an acute elevation greater than 2 points [17, 20]. In addition, patients younger than 18 years old, characterized as in-hospital death or with AKI occurring within 24 h of diagnosis of sepsis, or with missing weight information were excluded. After determining the stay_id of the study population, we extracted their relevant information using the Structured Query Language (SQL) Programming by Navicat Premium 11.2.7.0.

### Exposure

The exposure in our study was the trajectory of changes in the urine output at the first, second, third, and fourth six-hour interval after the diagnosis of sepsis. Urine output in this study was defined as the average urine output per kilogram of body weight per hour for each time period as follows: urine output in the first six hours = total urine output in the first six hours/(body weight × 6).

The LGMM model was used to classify the trajectory of changes in the urine output. A key factor in the generation of LGMM is that the number of potential classes should be specified. To select the best number of potential classes, we first set a quadratic growth model with a single class and then successively increase the number of classes to establish models corresponding to 2–6 classes. Indicators reflecting the goodness of fit of LGMM include log likelihood, entropy, and information criteria as follows: Akaike Information Criterion (AIC), Bayesian Information Criteria (BIC), and sample-adjusted BIC (SABIC) [21]. We determined the optimal number of classes according to the principle that the goodness of fit of a model is better when the information criterion is lower, and the log likelihood and entropy are higher. In addition, to ensure the statistical power of subsequent analysis, we limited the sample size of each class to no less than 1% of the total study population. Finally, the fitting effect of the model was evaluated by the means of the posterior probabilities in each class. The value of the posterior probability was between 0 and 1, and the closer it was to 1, the more accurate the classification was. In this study, we limited the mean of the posterior probability in each class to no less than 70%. The simplicity and clinical interpretability of the model were also considered.

### Outcome

The outcome of this study is AKI that occurs 24 h after the diagnosis of sepsis. The occurrence of AKI was an increase in the serum creatinine level or a decrease in the urine output as defined by the KDIGO as follows: an increase in serum creatinine by ≥ 0.3 mg/dL (≥ 26.5 µmol/L) within 48 h; an increase in serum creatinine to ≥ 1.5 times baseline within the previous 7 days; urine volume ≤ 0.5 mL/kg/h for 6 h [12, 22]. Follow-up began with the diagnosis of sepsis and ended at whichever was the earliest of: onset of AKI, in-hospital death, or discharge. In subsequent analyses, cases of in-hospital death or without AKI occurrence until discharge were regarded as censored in the Cox model. In the competing risk model, cases of in-hospital death were regarded as competing events, and cases without AKI occurrence until discharge were regarded as censored.

### Covariates

Any factors that might confuse the relationship between exposure and outcome were considered covariates and adjusted in subsequent studies. These factors included the demographic variables, disease severity scoring systems, laboratory test indicators, and treatment-related variables as follows: age, gender, ethnicity, first care unit type, Simplify Acute Physiological Scores II (SAPSII), Sequential Organ Failure Assessment (SOFA), Charlson comorbidity index, white blood cells (WBC), red blood cells (RBC), hemoglobin, red blood cell distribution width (RDW), platelet, sodium, potassium, chloride, bicarbonate, anion gap (AG), glucose, creatinine, blood urea nitrogen (BUN), usage of ventilator, use of vasopressor, and continuous renal replacement therapy (CRRT). For all measures taken multiple times during hospitalization, we used results from the first examination after the diagnosis of sepsis.

### Statistical analysis

Missing data were very common in the MIMIC-IV database. We used the “mice” package of R software to deal with the missing values of covariables by multiple imputation. In addition, to reduce information bias, no variables with a missing ratio of more than 10% were included in this study. Cases with a missing information ratio of more than 5% were excluded. Additional file 1: Fig. S1 shows the scenario of missing variables before imputation.

Ethnicity was divided into three categories, namely, white, black, and others. First care unit was also divided into three categories, namely, medical ICU (MICU)/surgical ICU (SICU), coronary care unit (CCU), and others. Continuous variables subject to normal distribution were described by means and standard deviations, and the distribution differences between trajectory classes were tested by ANOVA. Those variables that did not follow the normal distribution were described by the median and interquartile range, and the distribution differences between classes were analyzed by Kruskal–Wallis test. Categorical variables were described by frequency and percentage, and χ^2^-test or Fisher’s exact test was used to test the distribution differences between classes.

First, Kaplan–Meier (KM) method was used to draw a cumulative incidence curve to show the occurrence of AKI in patients with different trajectory classes, and log-rank test was used to compare the risk differences between classes. Then, four Cox proportional hazard models with increasing covariates were established to analyze the influence of the trajectory of changes in the urine output on the risk of AKI. Model 1 was univariate analysis without adjusting any covariates. In model 2, the age, gender, ethnicity, and first care unit were adjusted. In model 3, in addition to the covariables in model 2, Charlson comorbidity index, SAPSII, and SOFA were adjusted. In addition to adjusting the covariables in model 3, the WBC, RBC hemoglobin, RDW, platelet, sodium, chloride, bicarbonate, AG, glucose, creatinine, BUN, usage of ventilator, vasopressor, and CRRT were adjusted in model 4.

Patients who had died in the hospital would no longer experience AKI occurrence. Thus, in-hospital deaths could be considered as the competing events of AKI occurrence. Under such circumstances, the use of Cox proportional hazard models would treat in-hospital deaths as censored, which could lead to competing risk bias. Therefore, we also used Fine-Gray proportional subdistribution risk regression to construct the above four models to analyze the competing risk to evaluate the stability of the results. Similarly, cumulative incidence curve was also plotted using the cumulative incidence function (CIF), and differences in the risk of AKI between trajectory classes were also compared using the Gray’s test.

Finally, the doubly robust estimation method was used to infer the independent association between the trajectory of urine output change and the risk of AKI. Propensity scoring models of the above 23 covariables and trajectory classes were established using the multinominal logical regression and Extreme Gradient Boosting (XGBoost). The estimated propensity scores were used as weights to generate two cohorts of inverse probability of treatment weighting (IPTW), namely, pseudo population whose distribution of covariates is independent of trajectory classes [20, 23]. XGBoost is an integrated machine learning algorithm based on a decision tree, which adopts gradient boosting framework. The contribution of each covariate to the XGBoost model and multinominal logical regression was also shown. The standardized mean differences (SMDs) of the original cohort were compared with those of the inverse probability weighted cohorts to test whether IPTW reduced the imbalance in the distribution of covariates between trajectory classes. Similarly, cumulative incidence curves were also plotted in the IPTW cohorts and log-rank test was also done. Univariate Cox regression was performed on the weighted cohort, and the IPTW model was adjusted for the still unbalanced variables between trajectory classes (variables with SMD > 0.1), thus achieving double robust analysis.

A stratified analysis was also performed according to age (< 65 years and ≥ 65 years), gender (male, female), first care unit (MICU/SICU, CCU, and others), use of ventilator (no, yes), use of vasopressor (no, yes), CRRT (no, yes), Charlson comorbidity index (< 5, ≥ 5), SAPSII (< 35, ≥ 35), and SOFA (< 3, ≥ 3) to assess the potential modified effect. The potential interactions were also evaluated by adding a cross product term of trajectory class with the above stratification variables to the model.

A two-tailed p value less than 0.05 was considered statistically significant. All statistical analyses were performed using the R software (4.0.3). The R package used included DataExplorer, lattice, MASS, nnet, mice, dplyr, magrittr, data.table, tidyverse, tableone, survey, survival, survsim, Survminer, mstate, rms, cmprsk, foreign, Matching, lcmm, ipw, twang, and xgboost.

## Results

### LGMM analysis and baseline characteristics

The goodness of fit statistics of the LGMM models are shown in Table 1. AIC, BIC, and SABIC showed a decreasing trend from the one-class to five-class models, while the log likelihood was increasing. However, the change was opposite to the change in the six-class model. The entropy (> 0.9) of five-class model was lower than those of one-class to three-class models but higher than those of the four-class and six-class models. The sample proportion of the trajectory class with the minimum population of the five-class model was 1.469%, which also met the preset standard. In addition, one class in the six-class model had the sample proportion of 0%. Therefore, the five-class model was the best.

**Table 1:** Statistics for choosing the best number of classes

Number of classes	Log likelihood	AIC	BIC	SABIC	Entropy	%class1	%class2	%class3	%class4	%class5	%class6
1	− 56976.9	113961.8	113990.6	113977.9	1.0000000	100.000000					
2	− 53114.0	106244.1	106301.7	106276.2	0.9311908	8.997872	91.002128				
3	− 51704.9	103433.7	103520.1	103482.0	0.9430789	88.570271	4.731989	6.697740			
4	− 50503.1	101038.1	101153.3	101102.4	0.9150838	7.427298	82.095450	1.459114	9.018138		
5	− 49930.4	99900.8	100044.8	99981.2	0.9182471	7.285439	80.565407	7.123315	3.556591	1.469247	
6	− 49987.6	100023.2	100196.0	100119.7	0.8380689	9.038403	1.499645	0.000000	78.873239	9.585571	1.003141

AIC: Akaike information criterion; BIC: Bayesian information criteria; SABIC: sample-adjusted information criteria

**Fig. 1:**
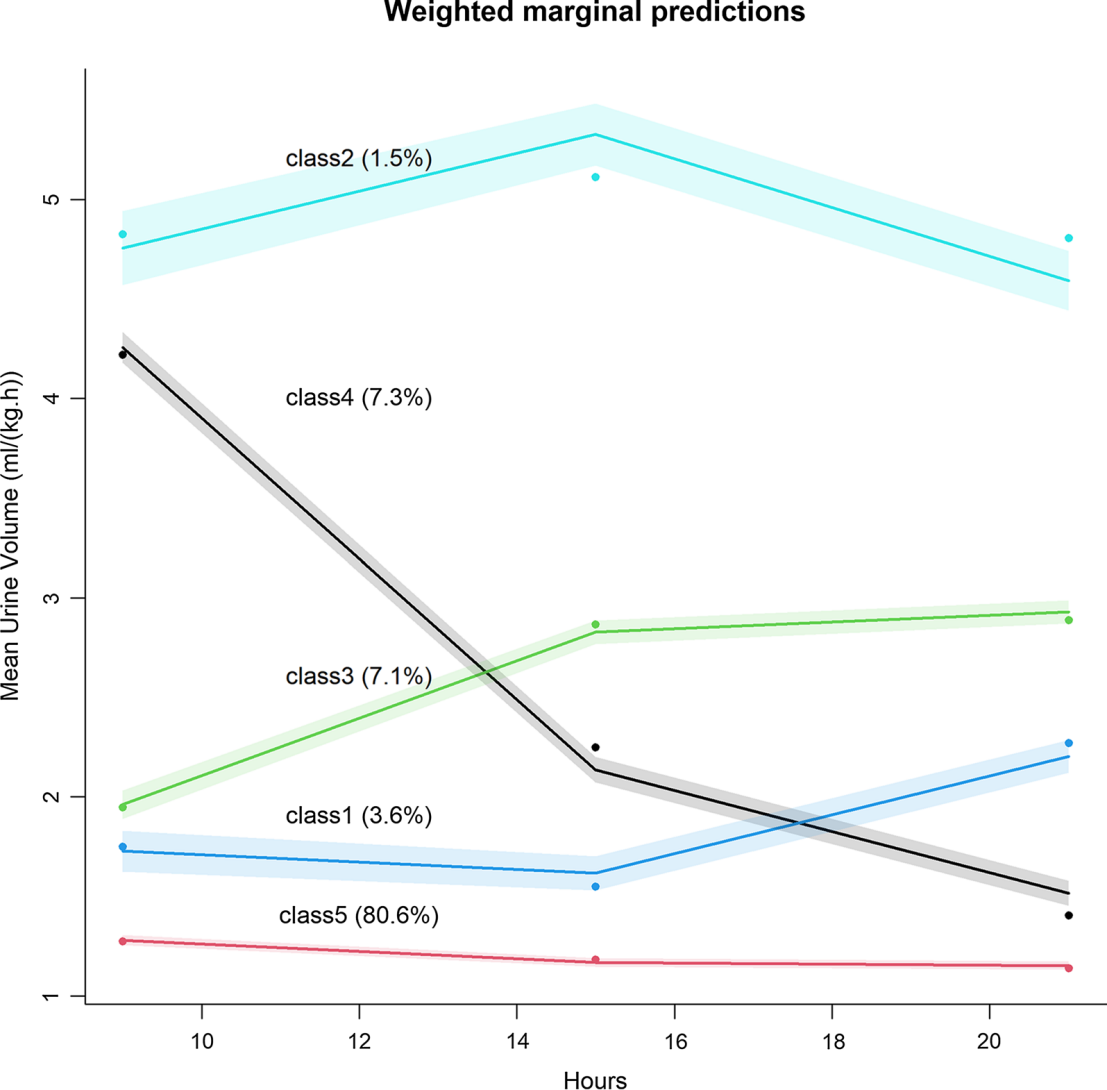
Five classes identified by trajectories of urine output. The shaded area indicates the 95% confidence interval for each mean trajectory. The percentages in the parentheses indicate the percentages of patients each class accounts for

The trajectory of the change in the urine output of the five-class model is shown in Fig. 1. Class 1 accounted for 3.6%, and the urine output was stable and then increased. Class 2 accounted for 1.5%, and the urine output was consistently at a high level, showing an inverted V-shaped trend of increasing first and then decreasing. Class 3 accounted for 7.1%, in which the urine output first rose and then stabilized. Class 4 accounted for 7.3%, in which the urine output was at a high level at the beginning and then decreased rapidly, with the final urine output level even lower than those of class 1 and class 3. Class 5 had the largest sample size, accounting for 80.6% of the total population, with the urine output at persistently low levels.

The means of the posterior probability of the five-class model were 87.56%, 97.08%, 82.49%, 83.06% and 95.74% for class 1, class 2, class 3, class 4, and class 5, respectively (Additional file 1: Table S1). The means were all higher than 70%, indicating the reliability of these results. The coefficients of five second-order functions of the five-class model are presented in Additional file 1: Table S2.

A total of 9,869 patients were included in this study, and their baseline characteristics are presented in Table 2. The incidence of AKI 24 h after the diagnosis of sepsis was 41.1%. The median age of the patients was 65 years old, and most of the patients were males (58.9%) and white (67.2%). Compared with patients in the other classes, the patients in class 2 were younger, less serious, and had fewer comorbidities, a lower proportion of ventilator and CRRT use, and the lowest incidence of AKI (17.2%). The specific urine output of each time period of the five classes is also shown in Table 2.

**Table 2:** Baseline characteristics of five classes

Variables	Overall	Class1	Class2	Class3	Class4	Class5	P-value
N	9869	351	145	703	719	7951	
Age, year	65.00 [54.00, 76.00]	61.00 [46.50, 71.00]	50.00 [36.00, 60.00]	60.00 [47.00, 71.00]	62.00 [51.00, 73.50]	66.00 [55.00, 77.00]	< 0.001
Gender (%)							
Male	5812 (58.9)	155 (44.2)	65 (44.8)	382 (54.3)	363 (50.5)	4847 (61.0)	< 0.001
Female	4057 (41.1)	196 (55.8)	80 (55.2)	321 (45.7)	356 (49.5)	3104 (39.0)	
Ethnicity (%)							
White	6631 (67.2)	224 (63.8)	64 (44.1)	438 (62.3)	455 (63.3)	5450 (68.5)	< 0.001
Black	873 (8.8)	31 (8.8)	23 (15.9)	63 (9.0)	66 (9.2)	690 (8.7)	
Others	2365 (24.0)	96 (27.4)	58 (40.0)	202 (28.7)	198 (27.5)	1811 (22.8)	
First_careunit (%)							
MICU/SICU	7113 (72.1)	245 (69.8)	121 (83.4)	512 (72.8)	413 (57.4)	5822 (73.2)	< 0.001
CCU	2433 (24.7)	95 (27.1)	20 (13.8)	163 (23.2)	284 (39.5)	1871 (23.5)	
Others	323 (3.3)	11 (3.1)	4 (2.8)	28 (4.0)	22 (3.1)	258 (3.2)	
Ventilator (%)							
No	3006 (30.5)	95 (27.1)	73 (50.3)	222 (31.6)	156 (21.7)	2460 (30.9)	< 0.001
Yes	6863 (69.5)	256 (72.9)	72 (49.7)	481 (68.4)	563 (78.3)	5491 (69.1)	
Vasopressor (%)							
No	6558 (66.5)	224 (63.8)	76 (52.4)	436 (62.0)	441 (61.3)	5381 (67.7)	< 0.001
Yes	3311 (33.5)	127 (36.2)	69 (47.6)	267 (38.0)	278 (38.7)	2570 (32.3)	
CRRT (%)							
No	9761 (98.9)	349 (99.4)	145 (100.0)	695 (98.9)	713 (99.2)	7859 (98.8)	0.517
Yes	108 (1.1)	2 (0.6)	0 (0.0)	8 (1.1)	6 (0.8)	92 (1.2)	
SAPSII	35.00 [28.00, 43.00]	34.00 [26.00, 40.00]	31.00 [23.00, 40.00]	33.00 [26.00, 42.00]	34.00 [27.00, 41.00]	36.00 [29.00, 43.00]	< 0.001
SOFA	3.00 [2.00, 4.00]	3.00 [2.00, 4.00]	3.00 [2.00, 4.00]	3.00 [2.00, 4.00]	3.00 [2.00, 4.00]	3.00 [2.00, 4.00]	0.011
Charlson comorbidity index	5.00 [3.00, 7.00]	5.00 [2.00, 7.00]	3.00 [1.00, 6.00]	5.00 [3.00, 7.00]	5.00 [3.00, 7.00]	6.00 [4.00, 8.00]	< 0.001
Laboratory tests							
WBC (k/uL)	11.00 [7.70, 15.30]	10.90 [7.55, 15.05]	11.30 [7.60, 16.30]	10.70 [7.30, 14.90]	11.30 [7.90, 15.10]	11.00 [7.70, 15.30]	0.354
RBC (m/uL)	3.38 [2.94, 3.84]	3.25 [2.95, 3.75]	3.42 [2.88, 3.95]	3.37 [2.95, 3.87]	3.36 [3.00, 3.84]	3.39 [2.94, 3.84]	0.427
Hemoglobin (g/dL)	10.10 [8.80, 11.50]	9.60 [8.60, 10.90]	10.10 [8.70, 11.60]	10.00 [8.80, 11.55]	10.20 [9.00, 11.50]	10.10 [8.80, 11.50]	0.015
RDW (%)	14.70 [13.60, 16.30]	14.70 [13.60, 16.35]	14.40 [13.50, 16.30]	14.60 [13.60, 16.50]	14.30 [13.40, 15.70]	14.70 [13.60, 16.30]	< 0.001
Platelet (k/uL)	178.00 [124.00, 249.00]	178.00 [118.50, 258.00]	196.00 [137.00, 258.00]	181.00 [124.00, 256.00]	171.00 [122.00, 232.50]	178.00 [124.00, 249.00]	0.296
Sodium (mEq/L)	139.00 [136.00, 141.00]	139.00 [135.00, 142.00]	140.00 [135.00, 142.00]	139.00 [136.00, 141.00]	139.00 [136.00, 141.00]	139.00 [136.00, 141.00]	0.579
Potassium (mEq/L)	4.10 [3.70, 4.50]	4.00 [3.60, 4.30]	3.80 [3.40, 4.30]	4.00 [3.60, 4.30]	4.00 [3.60, 4.40]	4.10 [3.70, 4.50]	< 0.001
Chloride (mEq/L)	106.00 [101.00, 109.00]	105.00 [101.50, 109.00]	107.00 [103.00, 112.00]	106.00 [101.00, 109.00]	106.00 [102.00, 110.00]	105.00 [101.00, 109.00]	0.001
Bicarbonate (mEq/L)	23.00 [20.00, 26.00]	23.00 [20.00, 26.00]	22.00 [18.00, 24.00]	23.00 [20.00, 25.00]	24.00 [21.00, 26.00]	23.00 [20.00, 26.00]	< 0.001
AG (mEq/L)	13.00 [11.00, 16.00]	13.00 [11.00, 15.50]	14.00 [11.00, 16.00]	14.00 [11.00, 16.00]	13.00 [11.00, 15.00]	13.00 [11.00, 16.00]	0.006
Glucose (mg/dL)	124.00 [103.00, 156.00]	123.00 [100.50, 159.00]	128.00 [103.00, 158.00]	119.00 [100.00, 150.00]	120.00 [103.00, 147.00]	125.00 [104.00, 157.00]	0.006
Creatinine (g/dL)	0.90 [0.70, 1.40]	0.80 [0.60, 1.10]	0.80 [0.60, 1.40]	0.90 [0.60, 1.40]	0.80 [0.60, 1.10]	1.00 [0.70, 1.40]	< 0.001
BUN (mg/dL)	18.00 [12.00, 32.00]	14.00 [9.00, 25.00]	12.00 [7.00, 24.00]	16.00 [9.00, 30.00]	14.00 [10.00, 21.00]	20.00 [13.00, 34.00]	< 0.001
Mean total urine output (mL/(kg h))	1.29 [0.95, 1.78]	2.30 [2.04, 2.66]	4.32 [3.97, 5.03]	2.38 [2.15, 2.75]	2.32 [2.04, 2.76]	1.15 [0.89, 1.46]	< 0.001
First six-hour urine output (mL/(kg h))	1.28 [0.83, 2.04]	1.58 [1.04, 2.12]	4.25 [3.28, 5.49]	1.98 [1.31, 2.53]	4.05 [3.54, 4.82]	1.12 [0.77, 1.67]	< 0.001
Second six-hour urine output (mL/(kg h))	1.16 [0.80, 1.77]	1.51 [0.97, 2.05]	4.99 [4.05, 6.20]	2.72 [2.15, 3.53]	2.04 [1.42, 2.74]	1.03 [0.75, 1.46]	< 0.001
Third six-hour urine output (mL/(kg h))	1.12 [0.78, 1.72]	2.05 [1.41, 2.85]	4.37 [3.36, 5.62]	2.88 [2.25, 3.59]	1.35 [0.98, 1.97]	1.01 [0.74, 1.44]	< 0.001
Fourth six-hour urine output (mL/(kg h))	1.12 [0.75, 1.75]	3.80 [3.39, 4.38]	3.67 [2.70, 4.90]	1.97 [1.44, 2.61]	1.45 [0.97, 2.19]	1.00 [0.70, 1.48]	< 0.001
AKI (%)							
No	5815 (58.9)	267 (76.1)	120 (82.8)	472 (67.1)	462 (64.3)	4494 (56.5)	< 0.001
Yes	4054 (41.1)	84 (23.9)	25 (17.2)	231 (32.9)	257 (35.7)	3457 (43.5)	
Endpoints (%)							
Alive	5515 (55.9)	251 (71.5)	115 (79.3)	446 (63.4)	448 (62.3)	4255 (53.5)	< 0.001
AKI	4054 (41.1)	84 (23.9)	25 (17.2)	231 (32.9)	257 (35.7)	3457 (43.5)	
In-hospital Death	300 (3.0)	16 (4.6)	5 (3.4)	26 (3.7)	14 (1.9)	239 (3.0)	
Follow-up time	4.21 [1.96, 7.72]	5.27 [3.19, 8.85]	5.56 [3.60, 9.58]	5.13 [2.74, 8.82]	4.82 [2.25, 8.17]	4.08 [1.83, 7.53]	< 0.001

MICU: medical intensive care unit; SICU: surgical intensive care unit; CCU: coronary care unit; CRRT: continuous renal replacement therapy; SAPSII: Simplify Acute Physiological Scores II; SOFA: Sequential Organ Failure Assessment; WBC: white blood cells; RBC: red blood cells; RDW: red blood cell distribution width; AG: anion gap; BUN: blood urea nitrogen; AKI: acute kidney injury

### Univariate and multivariate analyses

**Fig. 2:**
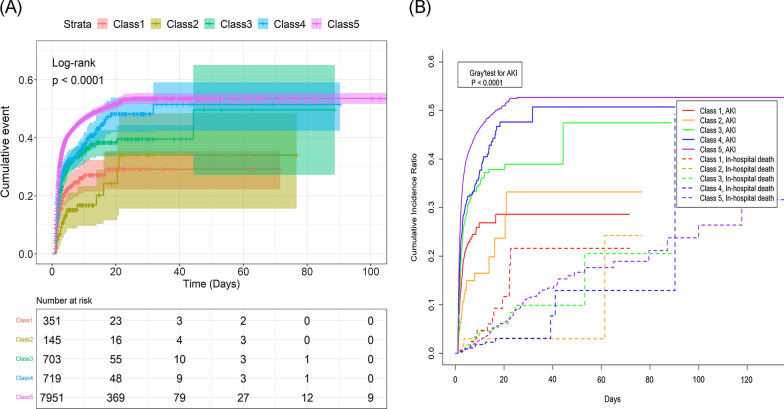
Cumulative incidence curves. **A** By Kaplan–Meier method, **B** by cumulative incidence function

The cumulative incidence curve drawn using the KM method is shown in Fig. 2A. The result of the log-rank test indicated that the risks of AKI were different among the five trajectory classes. The risk of class 5 was higher than those of the other classes at all time points. One interesting phenomenon was that the curve showed that the risk of AKI in class1 was higher than in class 2 before 20 days, and then reversed after 20 days. Therefore, we used the "ComparisonSurv" package of R software to test the difference of short-term and long-term risks between the two classes with a limit of 20 days. The results showed that there was no significant difference in short-term and long-term risks between the two classes. The cumulative incidence curve drawn using the CIF method Fig. 2B and the Gray’s test presented similar results.

The results of the Cox proportional hazard model are shown in Table 3. In the four models with different covariates adjusted, compared with class1, the hazard ratio (HR) of class 2 was lower than 1, but without statistical difference. Thus, class 2 did not have a significantly lower risk of AKI than class 1, but the other three classes had higher risks of AKI than class 1. In model 4 with the most adjusted covariates, HR (95% CI) values for class 3, class 4, and class 5 were 1.460 (1.137–1.875), 1.532 (1.197–1.961), and 2.232 (1.795–2.774), respectively. Hence, the order of the five types of AKI risks was class 5 > class 4 > class 3 > class 1 = class 2.

**Table 3:** Results of Cox proportional hazard models

Class	Model1		Model2		Model3		Model4	
	HR (95% CI)	P value	HR (95% CI)	P value	HR (95% CI)	P value	HR (95% CI)	P value
Class1	Reference		Reference		Reference		Reference	
Class2	0.693 (0.443–1.083)	0.107	0.759 (0.485–1.187)	0.227	0.750 (0.479–1.172)	0.207	0.844(0.540–1.321)	0.458
Class3	1.416 (1.103–1.818)	0.006	1.438 (1.120–1.846)	0.004	1.442 (1.123–1.852)	0.004	1.460 (1.137–1.875)	0.003
Class4	1.638 (1.280–2.095)	< 0.001	1.565 (1.223–2.002)	< 0.001	1.572 (1.228–2.011)	< 0.001	1.532 (1.197–1.961)	< 0.001
Class5	2.144 (1.727–2.663)	< 0.001	2.108 (1.696–2.619)	< 0.001	2.144 (1.726–2.664)	< 0.001	2.232 (1.795–2.774)	< 0.001

HR: hazard ratio; CI: confidence interval

From Table 2, the competing risk bias caused by in-hospital death in this study was small (by the end of follow-up, 41.1% of the patients had AKI occurrence, and only 3.0% of the patients died in the hospital). Therefore, the competing risk analysis using the Fine-Gray proportional subdistribution risk model showed results similar to those of the Cox proportional hazard regression (Table 4).

**Table 4:** Results of Fine-Gray proportional subdistribution hazard models

Class	Model1		Model2		Model3		Model4	
	HR (95% CI)	P value	HR (95% CI)	P value	HR (95% CI)	P value	HR (95% CI)	P value
Class1	Reference		Reference		Reference		Reference	
Class2	0.693 (0.449–1.070)	0.098	0.760 (0.492–1.173)	0.215	0.750 (0.486–1.158)	0.194	0.843 (0.552–1.287)	0.429
Class3	1.420 (1.112–1.812)	0.005	1.441 (1.129–1.840)	0.003	1.446 (1.134–1.845)	0.003	1.462 (1.150–1.859)	0.002
Class4	1.644 (1.291–2.096)	< 0.001	1.572 (1.233–2.005)	< 0.001	1.579 (1.239–2.013)	< 0.001	1.539 (1.212–1.955)	< 0.001
Class5	2.149 (1.738–2.658)	< 0.001	2.115 (1.709–2.618)	< 0.001	2.153 (1.740–2.664)	< 0.001	2.239 (1.815–2.761)	< 0.001

HR: hazard ratio; CI: confidence interval

### Double robust analysis

The SMDs of the original dataset, the IPTW dataset based on multinomial logistic regression, and the IPTW dataset based on the XGBoost are shown in Additional file 1: Fig. S2. The covariates between classes were well-balanced after IPTW, and XGBoost had a better effect than multinomial logistic regression. After the IPTW by multinomial logistic regression, the distribution of three covariables, namely, BUN, creatinine, and AG, remained unbalanced. By contrast, the distribution of only ethnicity was unbalanced after IPTW by XGBoost. In general, the IPTW dataset based on XGBoost also had lower SMDs compared with the IPTW dataset based on the multinomial logistic regression.

Additional file 1: Fig. S3 shows the degree of contribution of each covariable to the propensity score in the propensity score matching model based on XGBoost. They reflected the degree of influence of the different covariables on the classification or the degree of imbalance between classes. The top five variables with the highest contributions according to order were platelet, glucose, WBC, BUN, and RBC. The result of multinomial logical regression was shown in Additional file 1: Table S3. The cumulative incidence curves in IPTW cohorts were shown in Additional file 1: Fig. S4.

Table 5 presents the results of double robust analysis. No statistically significant difference was found between class 1 and class 3 in the risk of AKI, whether multinomial logistic regression or XGBoost was used, whether in univariate Cox analysis after IPTW, or multivariate Cox analysis in which only unbalanced covariates were adjusted or all covariates were adjusted. The risks of class 3, class 4, and class 5 were significantly higher than that of class 1, reflecting the stability of the results.

**Table 5:** Results of double robust analysis

Models	Class1	Class2		Class3		Class4		Class5	
		HR (95%CI)	P value	HR (95%CI)	P value	HR (95%CI)	P value	HR (95%CI)	P value
IPTW-Logistic									
Propensity score IPTW	Reference	0.959 (0.509–1.807)	0.897	1.611 (1.206–2.151)	0.001	1.510 (1.101–2.072)	0.011	2.364 (1.830–3.054)	< 0.001
Doubly robust with unbalanced covariates	Reference	0.950 (0.515–1.752)	0.870	1.598 (1.195–2.137)	0.002	1.514 (1.116–2.054)	0.008	2.342 (1.811–3.027)	< 0.001
Doubly robust with all covariates	Reference	0.951 (0.532–1.699)	0.865	1.554 (1.163–2.075)	0.003	1.502 (1.120–2.013)	0.007	2.388 (1.853–3.079)	< 0.001
IPTW-XGboost									
Propensity score IPTW	Reference	0.759 (0.411–1.402)	0.379	1.382 (1.034–1.849)	0.029	1.425 (1.063–1.912)	0.018	2.084 (1.613–2.693)	< 0.001
Doubly robust with unbalanced covariates	Reference	0.764 (0.412–1.418)	0.394	1.384 (1.035–1.851)	0.029	1.428 (1.064–1.917)	0.018	2.084 (1.612–2.694)	< 0.001
Doubly robust with all covariates	Reference	0.754 (0.424–1.343)	0.338	1.417 (1.059–1.896)	0.019	1.355 (1.011–1.817)	0.042	2.172 (1.676–2.816)	< 0.001

HR: hazard ratio; CI: confidence interval; IPTW: inverse probability of treatment weighting

### Subgroup analysis

Subgroup analysis was also conducted, and the results are shown in Additional file 1: Table S4. No statistical significance was found in the cross-product terms of all stratified variables with the trajectory class, indicating the absence of interaction. The correlation between the classification of the trajectory of urine output and the risk of AKI in the population of sepsis patients with different characteristics was consistent.

## Discussion

Urine output is one of the most important indicators in critically ill patients. Changes in urine output are closely related to physiological responses, changes in tissue perfusion, renal dysfunction, and clinical treatment [24]. This parameter is now widely used as one of the criteria for the diagnosis and staging of AKI [12]. Oliguria is also a cause of adverse after-effects in severe patients. As several studies have demonstrated very clearly, the duration of oliguria in the ICU patients is associated with the initiation of dialysis and an increased risk of death [25]. Urine output is a dynamic and continuous indicator. In this study, LGMM was used to study the correlation between the trajectory of urine output within 24 h and S-AKI. Various methods, such as competitive risk model and inverse probability weighting, were adopted to verify the results. All results showed that when the patient was diagnosed with sepsis, compared with patients whose urine output was stabilized and then rose (class 1), patients with consistently high urine output (class 2) did not have a higher risk of AKI. At the same time, patients’ whose urine output first increased and then stabilized (class 3), rapidly declined (class 4), and was persistently at low levels (class 5) had an increased risk of AKI. In general, the order of the five types of AKI risks was class 5 > class 4 > class 3 > class 1 = class 2. Moreover, among the five different 24-h urine volume trajectories, none of the patients has a urine volume ≤ 0.5 mL/kg/h, which is the most valuable point in this study, it can help clinicians identify the risk of AKI before patients meet the diagnostic criteria of AKI, so as to take corresponding clinical measures and interventions to prevent the occurrence of S-AKI and improve the prognosis of patients with sepsis.

Next, we will explain the results step by step, compared with class 1, the results of class 4 and class 5 were not surprising. The rapid drop in the urine level or a consistently low urine output indicated that such patients may not have received further treatment, or the urine output has continued to be low even after taking fluid rehydration therapy [26]. The pathophysiology of S-AKI involves complex processes including ischemia/reperfusion injury and inflammation [27]. Severe inflammatory responses in sepsis lead to endothelial failure, increased vascular permeability, and hypovolemia, resulting in renal perfusion and rapid decrease in urination [28]. The results demonstrated that early renal perfusion in patients with sepsis was closely related to the occurrence of AKI. In addition, studies in recent years have supported the view that organ dysfunction in these patients is not secondary only to hypoperfusion. Given the intense inflammatory response and microvascular dysfunction, the concentration of cytokines, chemokines, and complement fragments in the kidney area greatly increases [29], which may damage the renal tubules, leading to the deterioration of renal function and reduced urination. This phenomenon also supports that reduced urination is the result of early kidney injury, and such patients are at increased risk of AKI [27].

Interestingly, patients in class 3 had an increased risk of AKI compared with class 1. The possible mechanism may be related to the early treatment of patients with sepsis. Similar to the updated “1 h bundle” in the Surviving Sepsis Campaign [30], the rapid circulation of resuscitation through intravenous fluids is a key component of sepsis management [31]. Studies have demonstrated that positive fluid balance is associated with an increased risk of AKI and is a negative predictor of renal function recovery [32]. The effect of fluid resuscitation on renal injury stems from the high vulnerability of the kidney to hypoxic injury. In fact, with poor oxygen solubility of fluids, hemodilution reduces blood viscosity, promotes intra-renal shunt and heterogeneity, decreases capillary density, and increases hypoxia in the renal cortex and medulla [24]. The reasons for the low perfusion in patients are not only related to insufficient blood output but also to decreased systemic vascular resistance [33]. Therefore, attention should be given to the rational use of vasopressor. That is, the early urine output of sepsis patients increased in a certain trajectory, which may reflect excessive fluid replacement or unreasonable use of vasopressors, which are all related to the increased risk of AKI. However, the risk of class 3 is still slightly lower than that of class5 and class4, indicating that the risk of AKI in patients with positive response to clinical treatment measures is still lower than that in patients without treatment or no response to treatment measures. In the course of clinical treatment, early identification of sepsis patients' response to fluid replacement, and rational use of fluid replacement and vasopressors after initial resuscitation is of great significance [34].

## Strengths and limitations of the study

This study has several advantages. In addition to LGMM, which was used to explore and classify the trajectory of urine output for 24 h in patients with sepsis, Cox proportional hazard model, competing risk model, double robust estimation and other methods were used to analyze the influence of urine output trajectory on the occurrence of AKI in patients with sepsis. The results are reliable and stable, providing the basis for the clinical treatment of patients with sepsis. Another significant point is that compared with the criteria of AKI, the results of this study can help predict the risk of AKI before diagnosis, so as to help clinical treatment and decision-making. However, this study also had several limitations. Firstly, MIMIC-IV is a single-center database, and selection bias exists in this study, which limits the extrapolation of our conclusions. Secondly, our study only explored the risk of AKI in patients with sepsis after entering the ICU 24 h later, and did not study the population who did not enter the ICU, which is related to the MIMIC database missing information on patients who are not admitted to the ICU. Thirdly, urine volume is the result of a combination of many factors, all of which could not be taken into account in this study. Furthermore, our baseline table and results of multinomial logical regression and XGBoost show the imbalance and the degree of covariate among the trajectory classification. However, whether these factors affect the trajectory of the dynamic change in the urine output and the mechanism of action still need further discussion in the follow-up studies. Finally, we only discussed the influence of the trajectory in the changes in urine output of a single indicator on the occurrence of AKI. In future studies, the occurrence of disease can be predicted by combining the common trend of changes in other related indicators.

## Conclusion

The trajectory of urine output within 24 h of sepsis patients has a certain impact on the occurrence of AKI. Compared with patients whose urine output was stable at the early stage and slightly increased at the later stage, patients with urine output that first increased and then stabilized and those with urine output that decreased rapidly or remained at a low level had an increased risk of AKI. Therefore, in the early treatment of sepsis, close attention should be paid to changes in the patient's urine output to prevent the occurrence of S-AKI.

## Supplementary Information

**Additional file 1: Figure S1.** Data missing before multiple imputation. SOFA, Sequential Organ Failure Assessment; SAPSII, Simplify Acute Physiological Scores II; CRRT, continuous renal replacement therapy; AG, anion gap; BUN, blood urea nitrogen; RBC, red blood cells; WBC, white blood cells; RDW, red blood cell distribution width. **Figure S2.** SMD of covariable before and after IPTW. SMD, standardized mean difference; IPTW, inverse probability of treatment weighting; BUN, blood urea nitrogen; SAPSII, Simplify Acute Physiological Scores II; RDW, red blood cell distribution width; SOFA, Sequential Organ Failure Assessment; CRRT, continuous renal replacement therapy;; AG, anion gap; RBC, red blood cells; WBC, white blood cells. **Figure S3.** The contribution of each covariate to the XGBoost model. WBC, white blood cells; BUN, blood urea nitrogen; RBC, red blood cells; RDW, red blood cell distribution width; SAPSII, Simplify Acute Physiological Scores II; AG, anion gap; SOFA, Sequential Organ Failure Assessment; CRRT, continuous renal replacement therapy. **Figure S4.** Cumulative incidence curves of IPTW cohorts. (A) by multinomial logistic regression, (B) by XGBoost. **Table S1. ** Mean of posterior probabilities in each class. **Table S2. ** Fixed effects in the longitudinal five classes model. **Table S3.** Results of multinomial logistic regression. **Table S4. **Results of subgroup analysis

## Data Availability

The data were available on the MIMIC-IV website at https://mimic-iv.mit.edu/.
