# Supplementary material for: Influence of the trajectory of the urine output for 24 h on the occurrence of AKI in patients with sepsis in intensive care unit
Source: J Transl Med. 2021 Dec 20;19:518. doi: 10.1186/s12967-021-03190-w (PMC8686667; doi:10.1186/s12967-021-03190-w)
Supplement: Supplementary file 1 — Additional file 1: Figure S1. Data missing before multiple imputation. SOFA, Sequential Organ Failure Assessment; SAPSII, Simplify Acute Physiological Scores II; CRRT, continuous renal replacement therapy; AG, anion gap; BUN, blood urea nitrogen; RBC, red blood cells; WBC, white blood cells; RDW, red blood cell distribution width. Figure S2. SMD of covariable before and after IPTW. SMD, standardized mean difference; IPTW, inverse probability of treatment weighting; BUN, blood urea nitrogen; SAPSII, Simplify Acute Physiological Scores II; RDW, red blood cell distribution width; SOFA, Sequential Organ Failure Assessment; CRRT, continuous renal replacement therapy;; AG, anion gap; RBC, red blood cells; WBC, white blood cells. Figure S3. The contribution of each covariate to the XGBoost model. WBC, white blood cells; BUN, blood urea nitrogen; RBC, red blood cells; RDW, red blood cell distribution width; SAPSII, Simplify Acute Physiological Scores II; AG, anion gap; SOFA, Sequential Organ Failure Assessment; CRRT, continuous renal replacement therapy. Figure S4. Cumulative incidence curves of IPTW cohorts. (A) by multinomial logistic regression, (B) by XGBoost. Table S1. Mean of posterior probabilities in each class. Table S2. Fixed effects in the longitudinal five classes model. Table S3. Results of multinomial logistic regression. Table S4. Results of subgroup analysis [file 12967_2021_3190_MOESM1_ESM.docx]

Table S1. Mean of posterior probabilities in each class.

| **Class** | **probability1** | **probability2** | **probability3** | **probability4** | **probability5** |
| --- | --- | --- | --- | --- | --- |
| **class1** | 87.56% | 6.67% | 4.16% | 1.16% | 0.44% |
| **class2** | 0.89% | 97.08% | 1.43% | 0.59% | 0.00% |
| **class3** | 3.41% | 9.94% | 82.49% | 3.87% | 0.28% |
| **class4** | 1.27% | 8.25% | 7.14% | 83.06% | 0.27% |
| **class5** | 0.83% | 0.00% | 2.80% | 0.63% | 95.74% |

Table S2. Fixed effects in the longitudinal five classes model.

| **Items** | **Coefficient** | **Standard error** | **Wald statistic** | **p-value** |
| --- | --- | --- | --- | --- |
| **Intercept class1** | 7.31530 | 0.12236 | 59.785 | <0.001 |
| **Intercept class2** | 1.42056 | 0.02481 | 57.269 | <0.001 |
| **Intercept class3** | 0.13354 | 0.13089 | 1.020 | 0.308 |
| **Intercept class4** | 2.87235 | 0.15297 | 18.777 | <0.001 |
| **Intercept class5** | 3.94708 | 0.20603 | 19.158 | <0.001 |
| **poly1 class1** | -3.65826 | 0.10195 | -35.884 | <0.001 |
| **poly1 class2** | -0.17015 | 0.02245 | -7.578 | <0.001 |
| **poly1 class3** | 2.26269 | 0.12718 | 17.792 | <0.001 |
| **poly1 class4** | -1.58321 | 0.14428 | -10.973 | <0.001 |
| **poly1 class5** | 1.17611 | 0.18453 | 6.374 | <0.001 |
| **poly2 class1** | 0.56266 | 0.01901 | 29.593 | <0.001 |
| **poly2 class2** | 0.02550 | 0.00441 | 5.783 | <0.001 |
| **poly2 class3** | -0.44801 | 0.02477 | -18.086 | <0.001 |
| **poly2 class4** | 0.46087 | 0.02962 | 15.562 | <0.001 |
| **poly2 class5** | -0.29664 | 0.03617 | -8.200 | <0.001 |

Table S3. Results of multinomial logistic regression.

| **Variables** | **Class2** | | **Class3** | | **Class4** | | **Class5** | | **P-value** |
| --- | --- | --- | --- | --- | --- | --- | --- | --- | --- |
|  | **OR(95%CI)** | **P-value** | **OR(95%CI)** | **P-value** | **OR(95%CI)** | **P-value** | **OR(95%CI)** | **P-value** |  |
| **Age** | 0.971(0.955-0.987) | <0.001 | 0.996(0.985-1.006) | 0.420 | 1.002(0.992-1.013) | 0.657 | 1.018(1.009-1.027) | <0.001 | <0.001 |
| **Gender** |  |  |  |  |  |  |  |  | <0.001 |
| Male | Reference |  | Reference |  | Reference |  | Reference |  |  |
| Female | 0.958(0.634-1.448) | 0.839 | 0.697(0.533-0.913) | 0.009 | 0.840(0.642-1.100) | 0.205 | 0.519(0.414-0.651) | <0.001 |  |
| **Ethnicity** |  |  |  |  |  |  |  |  | 0.003 |
| White | Reference |  | Reference |  | Reference |  | Reference |  |  |
| Black | 2.302(1.225-4.326) | 0.010 | 1.075(0.673-1.719) | 0.762 | 1.270(0.796-2.028) | 0.316 | 1.051(0.708-1.560) | 0.806 |  |
| Others | 1.738(1.116-2.709) | 0.015 | 1.080(0.800-1.456) | 0.615 | 1.048(0.777-1.414) | 0.758 | 0.884(0.686-1.139) | 0.341 |  |
| **First_careunit** |  |  |  |  |  |  |  |  | <0.001 |
| MICU/SICU | Reference |  | Reference |  | Reference |  | Reference |  |  |
| CCU | 0.732(0.414-1.292) | 0.282 | 0.899(0.651-1.241) | 0.516 | 1.584(1.160-2.165) | 0.004 | 0.665(0.508-0.871) | 0.003 |  |
| Others | 0.702(0.400-1.234) | 0.219 | 1.206(0.737-1.974) | 0.456 | 1.266(0.757-2.119) | 0.369 | 1.003(0.683-1.475) | 0.986 |  |
| **Ventilator** |  |  |  |  |  |  |  |  | <0.001 |
| No | Reference |  | Reference |  | Reference |  | Reference |  |  |
| Yes | 0.308(0.196-0.484) | <0.001 | 0.837(0.614-1.142) | 0.263 | 1.034(0.750-1.426) | 0.838 | 0.947(0.727-1.233) | 0.685 |  |
| **Vasopressor** |  |  |  |  |  |  |  |  | <0.001 |
| No | Reference |  | Reference |  | Reference |  | Reference |  |  |
| Yes | 1.616(1.034-2.525) | 0.035 | 1.060(0.789-1.426) | 0.698 | 1.119(0.833-1.502) | 0.455 | 0.791(0.616-1.015) | 0.065 |  |
| **CRRT** |  |  |  |  |  |  |  |  | 0.233 |
| No | Reference |  | Reference |  | Reference |  | Reference |  |  |
| Yes | 0.235(0.234-0.236) | <0.001 | 2.130(1.222-3.712) | 0.008 | 1.606(0.877-2.941) | 0.125 | 2.560(1.709-3.835) | <0.001 |  |
| **SAPSII** | 1.010(0.988-1.032) | 0.375 | 1.001(0.987-1.015) | 0.867 | 1.004(0.991-1.019) | 0.532 | 0.995(0.983-1.007) | 0.382 | 0.070 |
| **SOFA** | 1.042(0.915-1.186) | 0.535 | 0.957(0.876-1.046) | 0.336 | 1.002(0.918-1.093) | 0.971 | 0.975(0.905-1.051) | 0.512 | 0.671 |
| **Charlson_comorbidity_index** | 0.999(0.904-1.103) | 0.979 | 1.033(0.970-1.102) | 0.312 | 1.030(0.965-1.099) | 0.374 | 1.065(1.009-1.125) | 0.023 | 0.035 |
| **Laboratory tests** |  |  |  |  |  |  |  |  |  |
| **WBC** | 0.993(0.968-1.018) | 0.565 | 0.993(0.978-1.009) | 0.395 | 0.998(0.983-1.014) | 0.824 | 1.005(0.993-1.017) | 0.441 | 0.107 |
| **RBC** | 0.465(0.241-0.898) | 0.023 | 0.645(0.426-0.976) | 0.038 | 0.644(0.422-0.983) | 0.041 | 0.680(0.482-0.959) | 0.028 | 0.105 |
| **Hemoglobin** | 1.442(1.142-1.822) | 0.002 | 1.287(1.108-1.496) | 0.001 | 1.253(1.076-1.459) | 0.004 | 1.286(1.134-1.458) | <0.001 | 0.001 |
| **RDW** | 1.013(0.921-1.115) | 0.786 | 1.048(0.984-1.117) | 0.145 | 0.975(0.912-1.042) | 0.457 | 1.017(0.963-1.074) | 0.542 | 0.174 |
| **Platelet** | 1.001(0.999-1.003) | 0.243 | 1.001(0.999-1.002) | 0.341 | 1.000(0.999-1.001) | 0.813 | 1.000(0.999-1.001) | 0.693 | 0.020 |
| **Sodium** | 1.102(0.988-1.230) | 0.083 | 1.056(0.981-1.138) | 0.149 | 0.953(0.885-1.027) | 0.208 | 1.043(0.980-1.111) | 0.185 | 0.001 |
| **Potassium** | 1.080(0.799-1.459) | 0.617 | 0.993(0.809-1.219) | 0.949 | 0.946(0.767-1.166) | 0.600 | 1.207(1.015-1.437) | 0.034 | <0.001 |
| **Chloride** | 0.936(0.839-1.045) | 0.240 | 0.943(0.875-1.015) | 0.120 | 1.069(0.992-1.152) | 0.079 | 0.964(0.905-1.026) | 0.246 | <0.001 |
| **Bicarbonate** | 0.882(0.786-0.991) | 0.034 | 0.925(0.856-1.001) | 0.052 | 1.069(0.988-1.156) | 0.097 | 0.945(0.885-1.010) | 0.093 | <0.001 |
| **AG** | 0.890(0.789-1.004) | 0.059 | 0.927(0.855-1.006) | 0.069 | 1.058(0.975-1.149) | 0.176 | 0.936(0.874-1.003) | 0.062 | <0.001 |
| **Glucose** | 1.000(0.999-1.001) | 0.982 | 1.000(0.999-1.001) | 0.987 | 0.999(0.998-1.001) | 0.350 | 0.999(0.998-1.000) | 0.152 | 0.378 |
| **Creatinine** | 1.086(0.828-1.425) | 0.552 | 1.035(0.845-1.267) | 0.742 | 0.924(0.729-1.171) | 0.514 | 0.882(0.735-1.058) | 0.175 | 0.025 |
| **BUN** | 0.994(0.977-1.011) | 0.493 | 1.005(0.995-1.016) | 0.326 | 0.999(0.987-1.010) | 0.808 | 1.014(1.005-1.024) | 0.003 | <0.001 |

Abbreviations: MICU, medical intensive care unit; SICU, surgical intensive care unit; CCU, coronary care unit; CRRT, continuous renal replacement therapy; SAPSII, Simplify Acute Physiological Scores II; SOFA, Sequential Organ Failure Assessment; WBC, white blood cells; RBC, red blood cells; RDW, red blood cell distribution width; AG, anion gap; BUN, blood urea nitrogen.

Table S4. Results of subgroup analysis.

| **Subgroups** | **No.of aki/No.of patients** | **class1** | **class2** | **class3** | **class4** | **class5** | **P for interaction** |
| --- | --- | --- | --- | --- | --- | --- | --- |
| **Age** |  |  |  |  |  |  |  |
| <65 | 1838/4764 | Reference | 0.984(0.585-1.655) | 1.505(1.068-2.122) | 1.612(1.147-2.264) | 2.473(1.835-3.333) | 0.697 |
| ≥65 | 2216/5105 | Reference | 0.569(0.203-1.593) | 1.419(0.983-2.047) | 1.455(1.016-2.085) | 2.024(1.473-2.782) |  |
| **Gender** |  |  |  |  |  |  |  |
| Male | 2399/5812 | Reference | 1.018(0.530-1.956) | 1.453(1.006-2.099) | 1.619(1.122-2.337) | 2.281(1.645-3.162) | 0.954 |
| Female | 1655/4057 | Reference | 0.735(0.397-1.361) | 1.485(1.052-2.095) | 1.452(1.038-2.030) | 2.181(1.628-2.922) |  |
| **First_careunit** |  |  |  |  |  |  |  |
| MICU/SICU | 2782/7113 | Reference | 0.764(0.448-1.304) | 1.280(0.935-1.753) | 1.545(1.129-2.115) | 2.176(1.659-2.853) | 0.63 |
| CCU | 1118/2433 | Reference | 1.185(0.486-2.889) | 1.919(1.227-3.003) | 1.560(1.012-2.405) | 2.341(1.580-3.468) |  |
| Others | 154/323 | Reference | 0.836(0.087-8.051) | 1.351(0.421-4.334) | 1.096(0.337-3.563) | 1.680(0.587-4.810) |  |
| **Ventilator** |  |  |  |  |  |  |  |
| No | 620/3006 | Reference | 0.870(0.261-2.905) | 1.551(0.710-3.388) | 1.814(0.805-4.085) | 2.667(1.322-5.378) | 0.684 |
| Yes | 3434/6863 | Reference | 0.890(0.548-1.444) | 1.457(1.118-1.898) | 1.506(1.162-1.952) | 2.186(1.739-2.749) |  |
| **Vasopressor** |  |  |  |  |  |  |  |
| No | 2280/6558 | Reference | 0.942(0.470-1.890) | 1.809(1.265-2.587) | 1.633(1.141-2.337) | 2.364(1.720-3.249) | 0.181 |
| Yes | 1774/3311 | Reference | 0.776(0.432-1.396) | 1.167(0.818-1.664) | 1.452(1.032-2.043) | 2.159(1.601-2.913) |  |
| **CRRT** |  |  |  |  |  |  |  |
| No | 3951/9761 | Reference | 0.853(0.545-1.337) | 1.455(1.129-1.876) | 1.534(1.195-1.969) | 2.246(1.803-2.800) | NA |
| Yes | 103/108 | Reference | NA | 1.452(0.245-8.619) | 0.743(0.124-4.462) | 1.466(0.316-6.809) |  |
| **Charlson_comorbidity_index** |  |  |  |  |  |  |  |
| <5 | 1372/3726 | Reference | 1.135(0.626-2.056) | 1.692(1.146-2.497) | 1.636(1.115-2.400) | 2.491(1.775-3.496) | 0.691 |
| ≥5 | 2682/6143 | Reference | 0.617(0.303-1.257) | 1.319(0.951-1.830) | 1.455(1.053-2.009) | 2.088(1.572-2.774) |  |
| **SAPSII** |  |  |  |  |  |  |  |
| <35 | 1622/4702 | Reference | 1.073(0.579-1.988) | 1.726(1.189-2.505) | 1.500(1.029-2.186) | 2.366(1.703-3.288) | 0.318 |
| ≥35 | 2432/5167 | Reference | 0.662(0.343-1.278) | 1.244(0.887-1.746) | 1.513(1.091-2.099) | 2.096(1.567-2.802) |  |
| **SOFA** |  |  |  |  |  |  |  |
| <3 | 1490/3926 | Reference | 0.709(0.294-1.705) | 1.415(0.933-2.148) | 1.539(1.013-2.340) | 2.144(1.489-3.087) | 0.988 |
| ≥3 | 2564/5943 | Reference | 0.909(0.538-1.536) | 1.485(1.085-2.031) | 1.537(1.131-2.087) | 2.272(1.732-2.982) |  |

Abbreviations: MICU, medical intensive care unit; SICU, surgical intensive care unit; CCU, coronary care unit; CRRT, continuous renal replacement therapy; SAPSII, Simplify Acute Physiological Scores II; SOFA, Sequential Organ Failure Assessment.

Figure S1


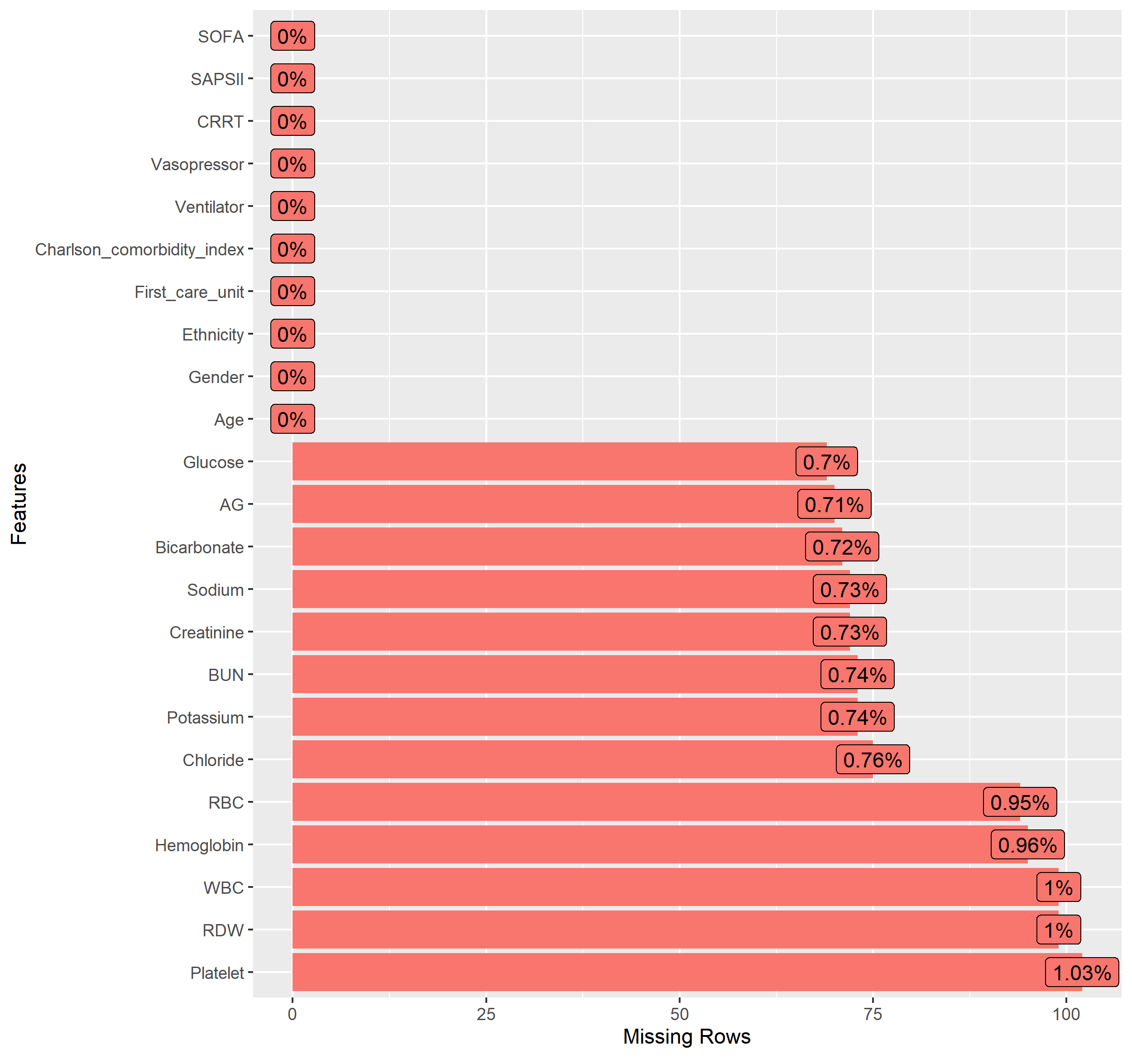


Figure S2


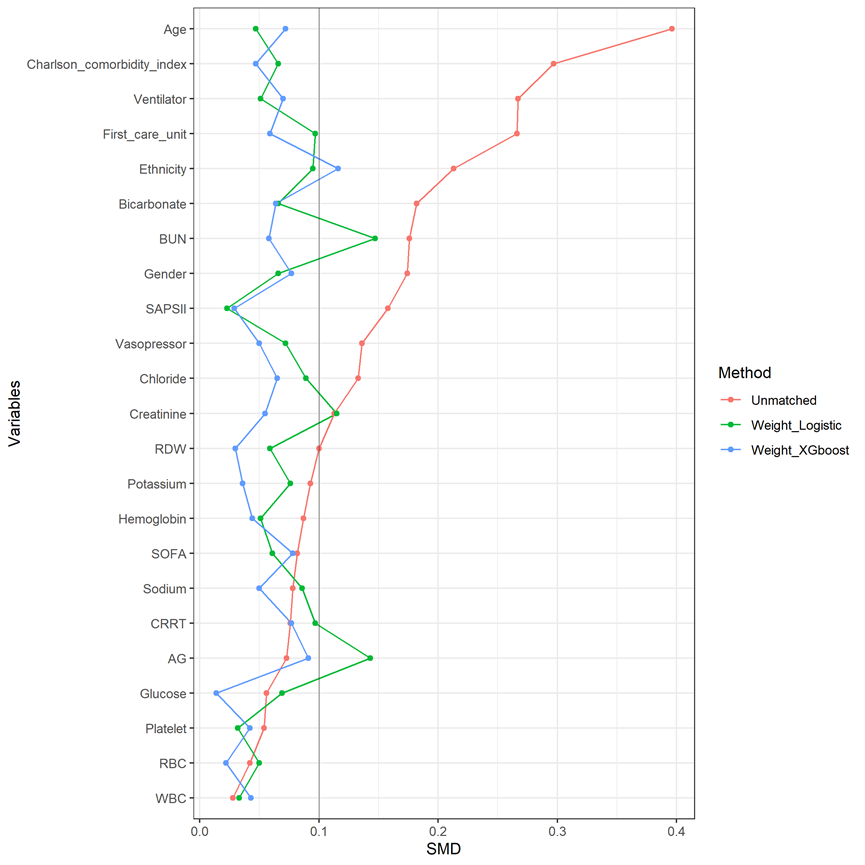


Figure S3


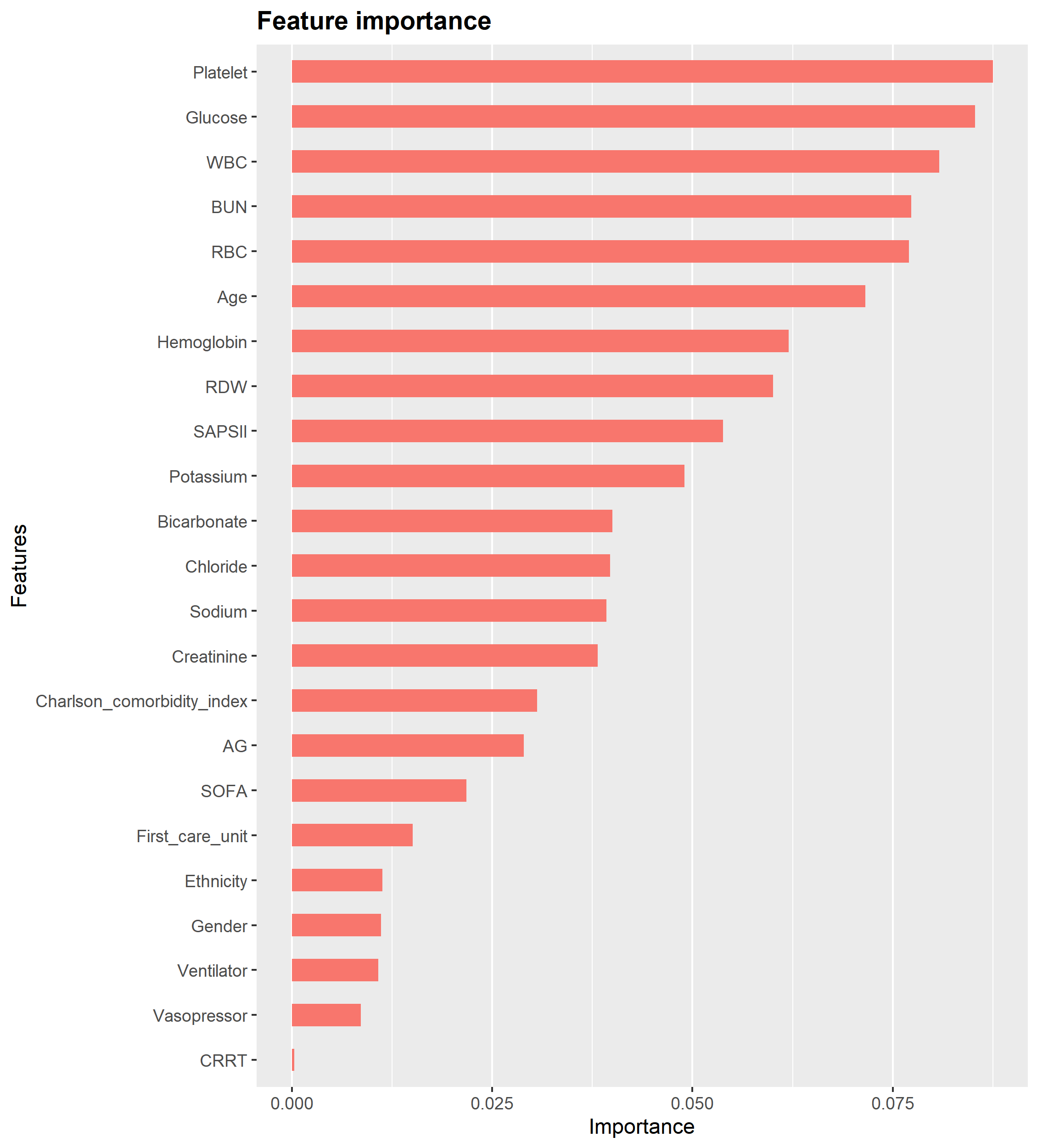


Figure S4


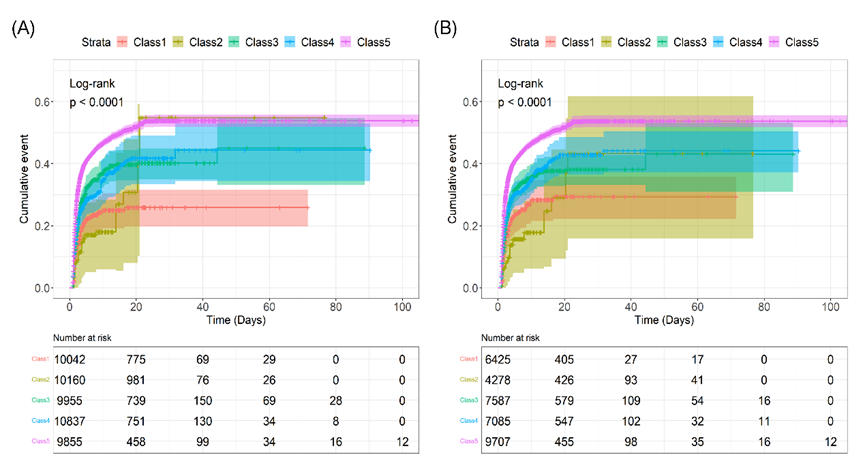


**The main R code of XGBoost and LGMM models**

**###XGBoost**

mnps.AOD<-mnps(class~age+gender+ethnicity+first_careunit+charlson_comorbidity_index+vent+vasop+crrt+sapsii+sofa_score+Bicarbonate+Creatinine+Glucose+Potassium+Urea.Nitrogen+Anion.Gap+Chloride+Sodium+Hemoglobin+Platelet.Count+RDW+Red.Blood.Cells+White.Blood.Cells,data = mydata,estimand = "ATE",version = "xgboost",verbose = FALSE,stop.method = c("es.mean"),n.trees = 5000)

w<- get.weights(mnps.AOD,stop.method = "es.mean")

mod4=coxph(Surv(akisofatimeday,aki)~as.factor(class),data=mydata,weight=w)

summary(mod4)

**###LGMM**

m1 <- hlme(uow ~ poly(hour, degree = 2, raw = TRUE),

subject = 'stay_id', ng = 1,

data = mydata)

...

m7 <- hlme(uow ~ poly(hour, degree = 2, raw = TRUE),

mixture = ~ poly(hour, degree = 2, raw = TRUE),

subject = 'stay_id', ng = 7,

data = mydata)

plot(m5, which = "fit", var.time = "hour", break.times=4,

bty = "l", ylab = "Mean Urine Volume (ml/(kg.h))",

xlab = "Hours", lwd = 2,

marg = T,legend=NULL,shades = T)

text(12,4,paste(colnames(postprob(m5)[[1]])[4],' (',round(postprob(m5)[[1]][2,1],1),'%',')',sep = ''))

text(12,1.4,paste(colnames(postprob(m5)[[1]])[5],' (',round(postprob(m5)[[1]][2,2],1),'%',')',sep = ''))

text(12,2.6,paste(colnames(postprob(m5)[[1]])[3],' (',round(postprob(m5)[[1]][2,3],1),'%',')',sep = ''))

text(12,1.9,paste(colnames(postprob(m5)[[1]])[1],' (',round(postprob(m5)[[1]][2,4],1),'%',')',sep = ''))

text(12,5.2,paste(colnames(postprob(m5)[[1]])[2],' (',round(postprob(m5)[[1]][2,5],1),'%',')',sep = ''))

summary(m5)

summarytable(m1,m2,m3,m4,m5,m6,which=c("G", "loglik", "conv", "npm", "AIC", "BIC", "SABIC", "entropy", "%class"))

postprob(m5)

dtclass <- cbind(m3$pprob[,1:2],m4$pprob[,2],m5$pprob[,2],m6$pprob[,2])
